# Supplementary material for: Linear and nonlinear chromatic integration in the mouse retina
Source: Nat Commun. 2021 Mar 26;12:1900. doi: 10.1038/s41467-021-22042-1 (PMC7997992; doi:10.1038/s41467-021-22042-1)
Supplement: Supplementary file 1 — Supplementary Information [file 41467_2021_22042_MOESM1_ESM.pdf]

Linear and nonlinear chromatic integration in the mouse retina

Mohammad Hossein Khani[1, 2, 3] * and Tim Gollisch[1, 2] *

[1] University Medical Center Göttingen, Dept. of Ophthalmology, 37073 Göttingen, Germany
[2] Bernstein Center for Computational Neuroscience, 37073 Göttingen, Germany
[3] International Max Planck Research School for Neuroscience, 37077 Göttingen, Germany

* Correspondence: mhkhanni@gmail.com (M.H.K.), tim.gollisch@med.uni-goettingen.de (T.G.).

Supplementary Materials

List of supplementary figures:

- Supplementary Figure1: Visual stimulation of mouse retina with a UV-green projector.
- Supplementary Figure 2: Sample responses of UV-selective and color-opponent cells to the chromatic integration stimulus.
- Supplementary Figure 3: Chromatic nonlinearity of the nonlinear On-Off cells is driven by their Off responses.
- Supplementary Figure 4: Responses of chromatically linear and nonlinear On-Off cells.
- Supplementary Figure 5: Nonlinear cells were found predominantly in the ventral region of the mouse retina.
- Supplementary Figure 6: Chromatic integration properties of direction-selective and orientation-selective ganglion cells.

Supplementary Figures:

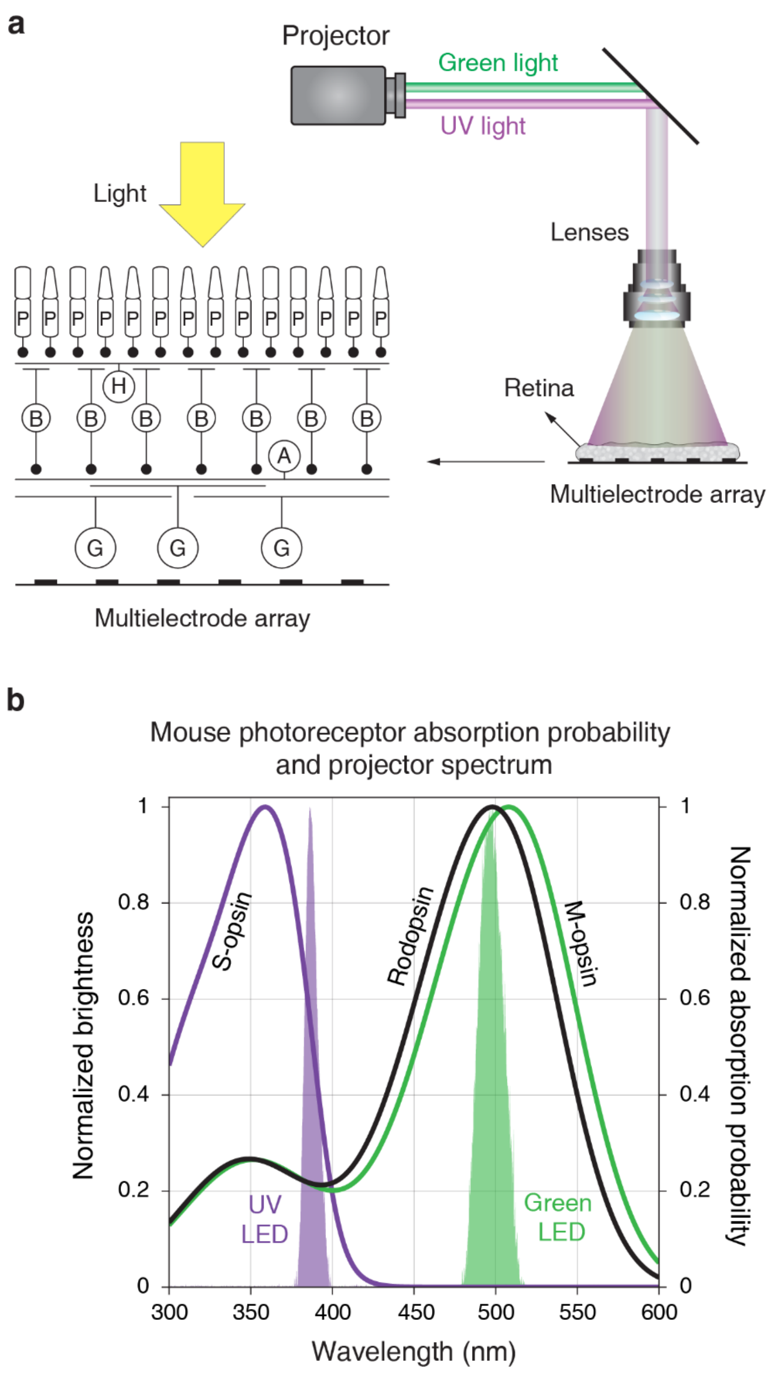

Supplementary Figure 1: Visual stimulation of mouse retina with a UV-green projector. a, Schematic view of the experimental setup. The mouse retina was mounted over a multielectrode array, and dichromatic, UV-green stimuli were displayed onto to the retina from above. **b**, Emission spectra of the LEDs from the projector used in this study (filled areas) and spectral sensitivities[1] of mouse photoreceptor opsins.

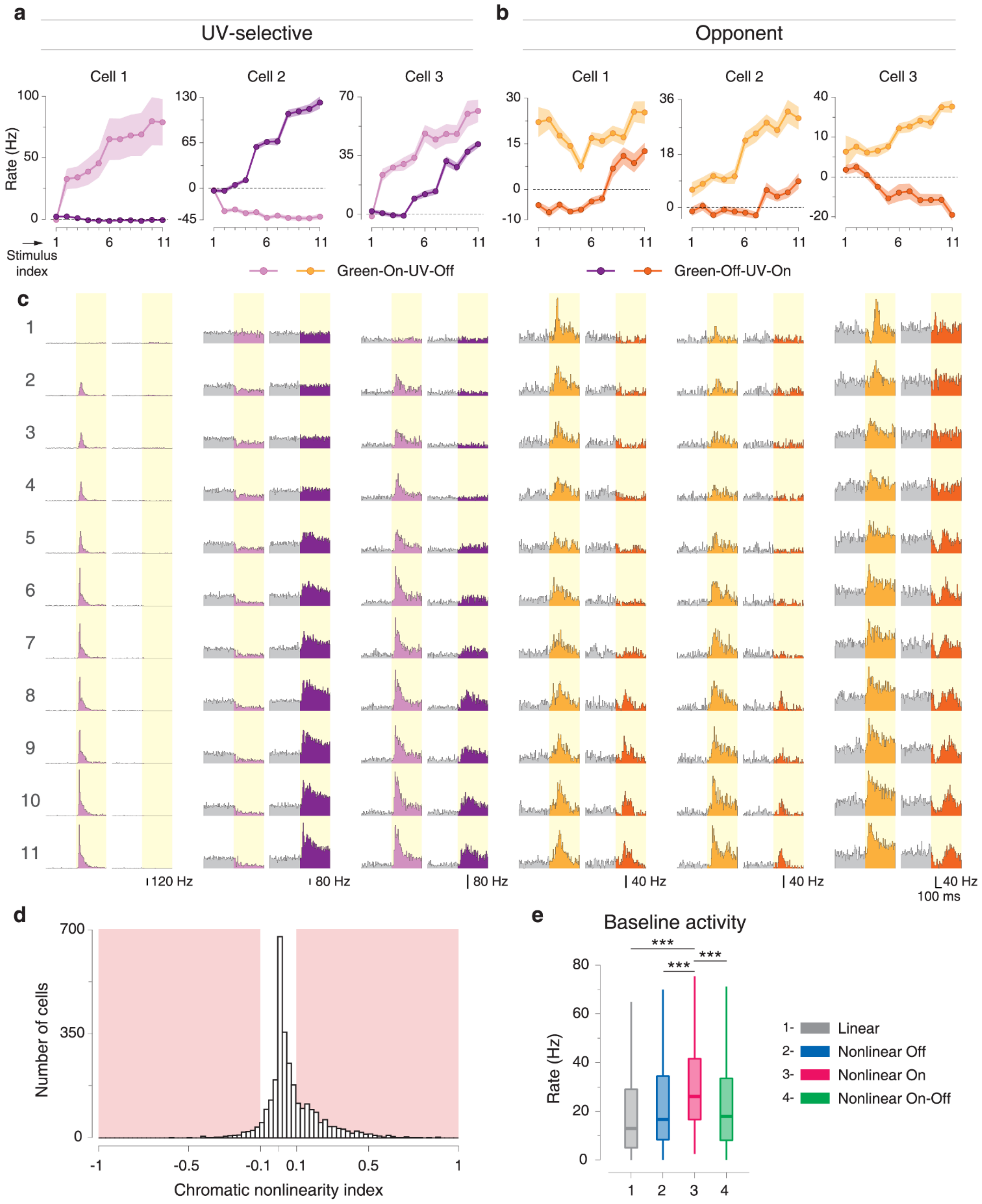

Supplementary Figure 2: Sample responses of UV-selective and color-opponent cells to the chromatic integration stimulus. a, Examples of UV-selective On, Off, and On-Off ganglion cells. These cells responded strongly to UV stimuli, but were unaffected by pure green stimuli (stimulus index 1). **b**, Three examples of color-opponent cells. These cells responded preferentially to the stimuli in one of the two sets, either green-On-UV-Off or green-Off-UV-On. Shaded regions around the curves show mean±SEM. **c**, PSTHs from the responses of the cells in **a-b** to all contrast combinations in the chromatic

integration stimulus. **d**, Distribution of the chromatic nonlinearity index of all recorded cells (n=2975). Cells with chromatic nonlinearity index >0.1 or < -0.1 were considered as nonlinear cells (shaded region, n=1039 out of 2975 cells totally recorded). **e**, Comparison of average baseline activity between linear (n=1936) and different classes of nonlinear cells (nonlinear Off cells: n=328, nonlinear On cells: n=144, and nonlinear On-Off cells: n=567). Nonlinear On cells showed a comparatively high level of baseline activity (two-sided Kruskal-Wallis test, d.f.=3, $p=5.1x10^{-18}$ with post-hoc analysis of mean ranks with Bonferroni correction, Statistics summary: *** p<0.001). the central line and the box mark the median and the interquartile range (IQR) from first to third quartile, respectively, and whiskers extend to the maximum and minimum within the central range of 1.5 x IQR.

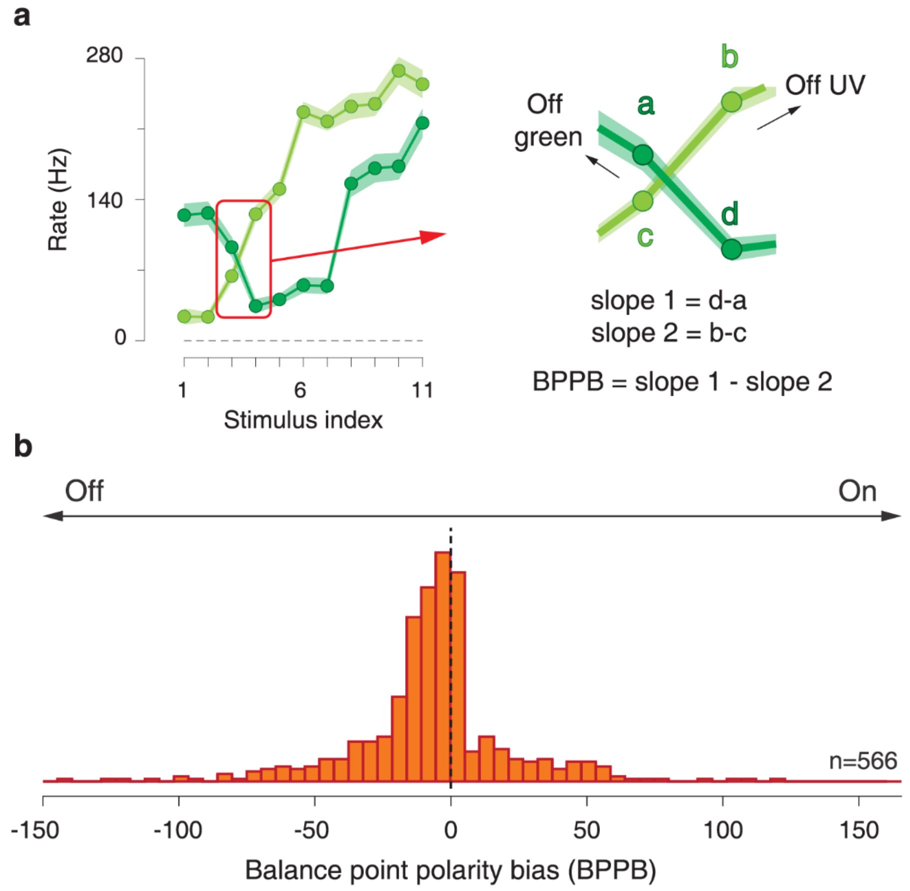

Supplementary Figure 3: Chromatic nonlinearity of the nonlinear On-Off cells is driven by their Off responses. a, Measurement of balance point polarity bias (BPPB) for chromatically nonlinear On-Off cells. The BPPB measures which contrast polarity governs the responses at the crossing point of the chromatic integration curves. It is defined as the difference in the slopes of the curves at the crossing point. Error bands show mean±SEM. **b**, Distribution of BPPB values. For the majority of chromatically nonlinear On-Off cells, the BPPB was negative, indicating that the nonlinearity of chromatic integration is driven by Off responses.

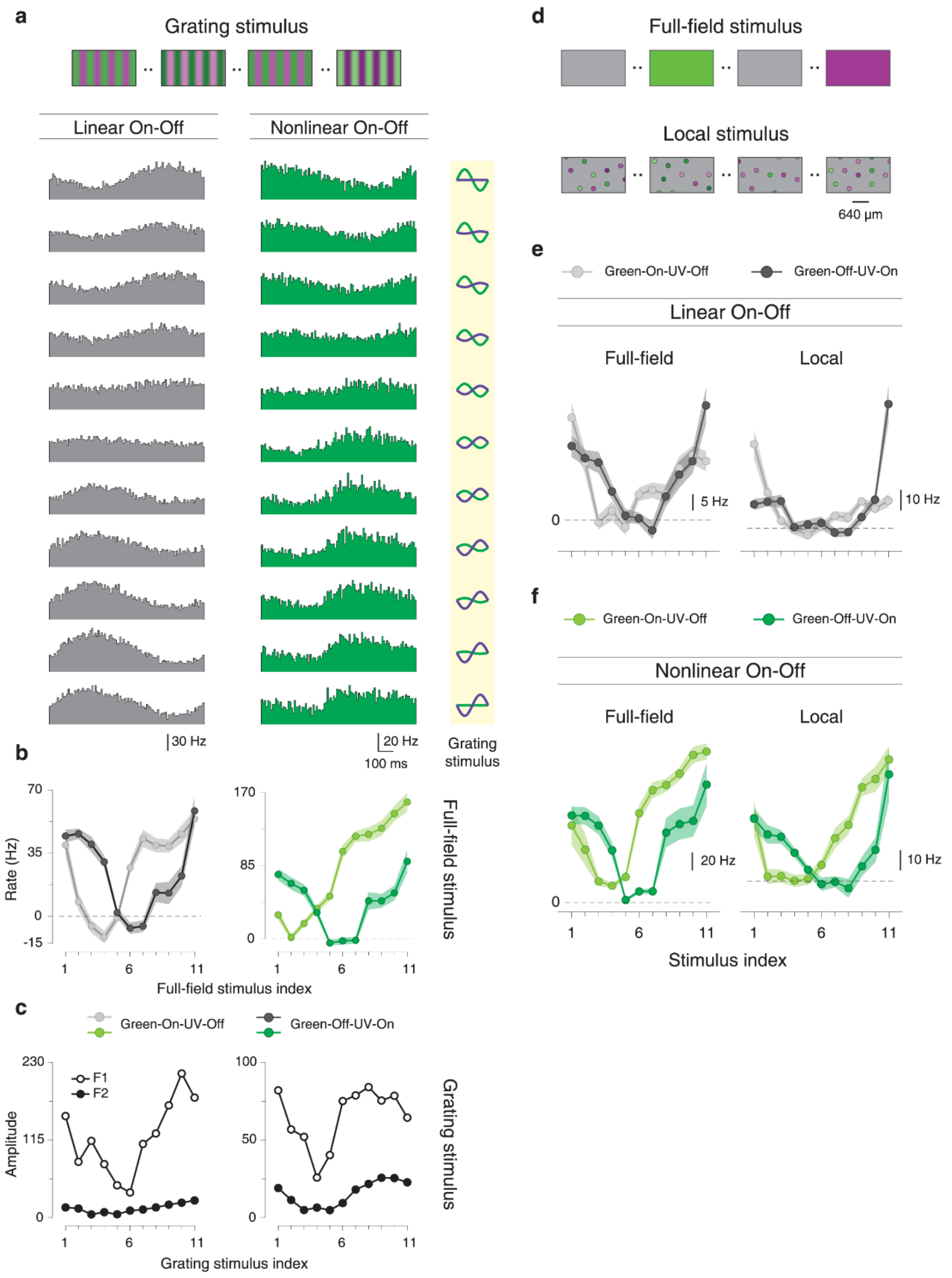

Supplementary Figure 4: Responses of chromatically linear and nonlinear On-Off cells. **a**, Responses of a linear On-Off and a nonlinear On-Off cell to the grating stimulus, revealing similar response profiles under grating stimulation. **b**, Responses of the cells in **a** to the spatially homogenous chromatic integration stimulus. Shaded regions around the curves show mean±SEM. **c**, Amplitudes of first and second harmonics measured from the responses of the cells to the grating stimulus in **a**. **d**, Schematic view of the full-field and the local chromatic integration stimuli. **e**, Chromatic integration curves of a linear On-Off cell for full-field and local stimulation. **f**, Chromatic integration curves of a nonlinear On-Off cell for full-field and local stimulation, indicating that the cell became linear under local stimulation.

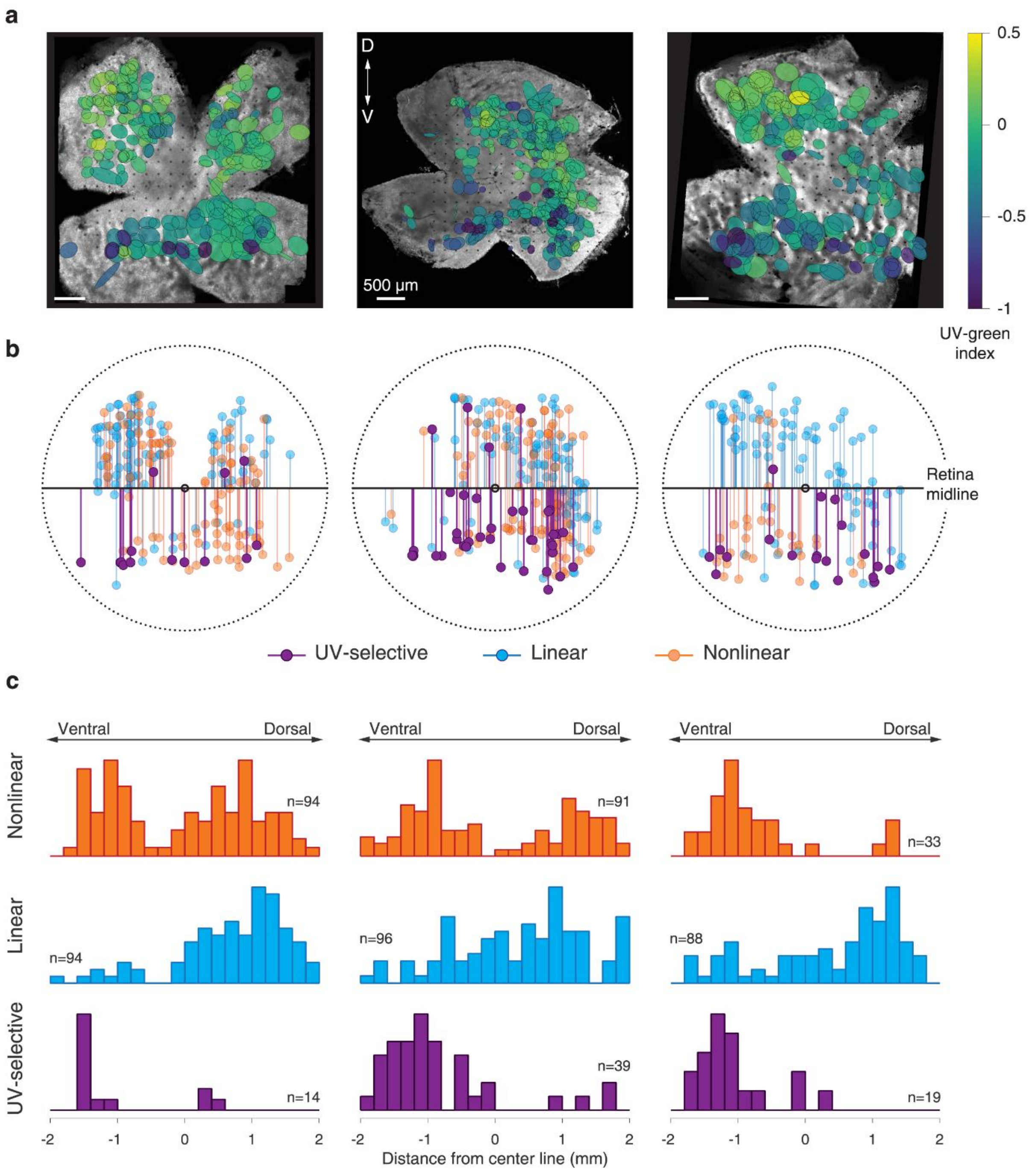

Supplementary Figure 5: Nonlinear cells were found predominantly in the ventral region of the mouse retina. a, Three retinas with overlaid spatial receptive fields of recorded ganglion cells. The receptive field colors show the UV-green index for each cell. **b**, Distances from the receptive field center of each cell to the horizontal midline of the retina. **c**, Distribution of receptive field distances from the midline for chromatically nonlinear, chromatically linear, and UV-selective cells from the retinas shown in **a**. In each case, the ratio of chromatically nonlinear to chromatically linear cells is higher in the ventral retina than in the dorsal retina.

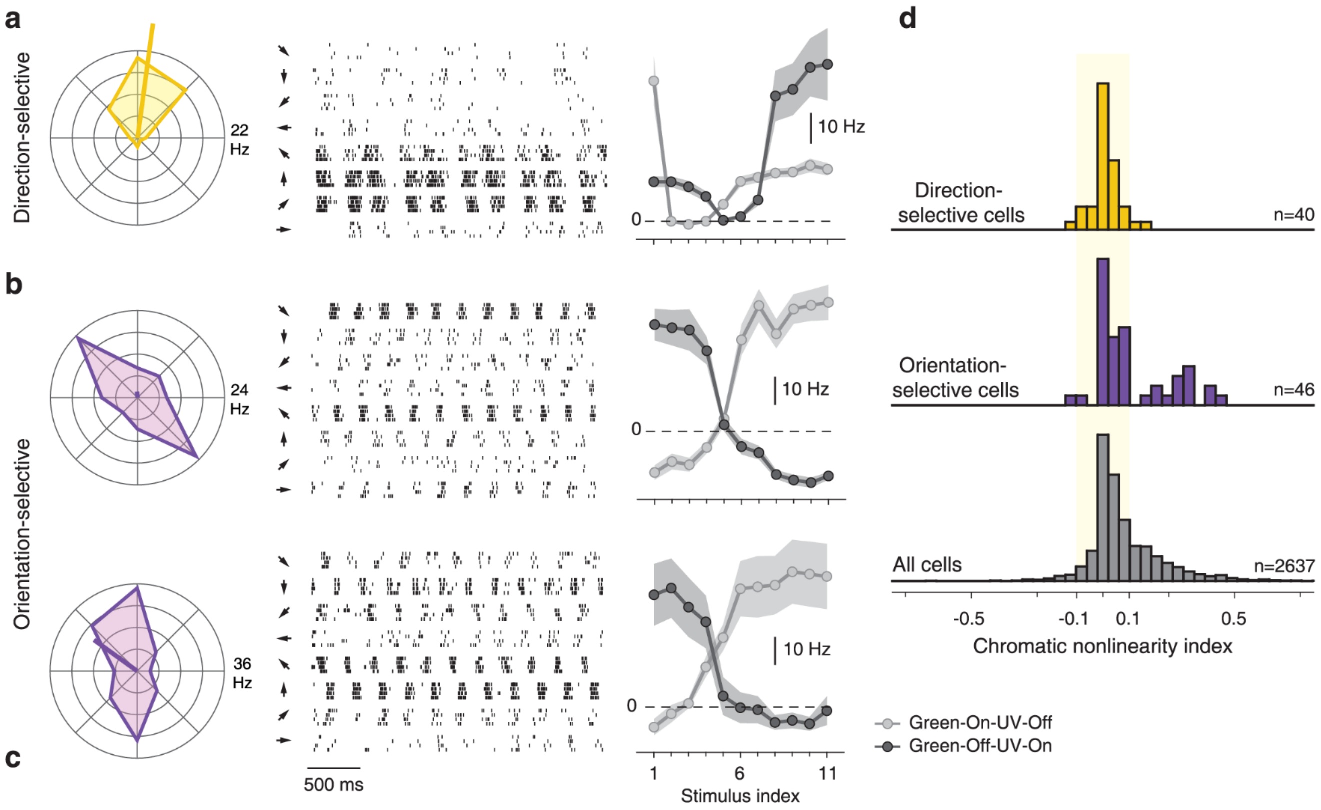

Supplementary Figure 6: Chromatic integration properties of direction-selective and orientation-selective ganglion cells. a, Responses of a sample On-Off direction-selective (DS) ganglion cell to a drifting grating stimulus. The tuning curve is shown on the left, raster plot in the center, and the chromatic integration curve on the right. **b**, same as **a**, but for a sample chromatically linear orientation-selective (OS) ganglion cell. **c**, same as **b**, but for a sample chromatically nonlinear OS ganglion cell. Shaded grey regions around the chromatic integration curves show mean±SEM. **d**, Distributions of chromatic nonlinearity indices for DS and OS cells. The data show that nearly all the measured DS cells were chromatically linear, whereas a sizable portion of OS cells were chromatically nonlinear.

Supplementary References

1. Govardovskii VI, Fyhrquist N, Reuter T, Kuzmin DG, Donner K. In search of the visual pigment template. *Vis Neurosci* **17**, 509-528 (2000).
