# Supplementary material for: Linear and nonlinear chromatic integration in the mouse retina
Source: Nat Commun. 2021 Mar 26;12:1900. doi: 10.1038/s41467-021-22042-1 (PMC7997992; doi:10.1038/s41467-021-22042-1)
Supplement: Supplementary file 3 — Reporting Summary [file 41467_2021_22042_MOESM3_ESM.pdf]

nature research

Corresponding author(s): Mohammad Khani
Tim Gollisch

Last updated by author(s): Feb 20, 2021

Reporting Summary

Nature Research wishes to improve the reproducibility of the work that we publish. This form provides structure for consistency and transparency in reporting. For further information on Nature Research policies, see our Editorial Policies and the Editorial Policy Checklist.

Statistics

For all statistical analyses, confirm that the following items are present in the figure legend, table legend, main text, or Methods section.

n/a	Confirmed	
☐	☒	The exact sample size (*n*) for each experimental group/condition, given as a discrete number and unit of measurement
☐	☒	A statement on whether measurements were taken from distinct samples or whether the same sample was measured repeatedly
☐	☒	The statistical test(s) used AND whether they are one- or two-sided<br>*Only common tests should be described solely by name; describe more complex techniques in the Methods section.*
☒	☐	A description of all covariates tested
☐	☒	A description of any assumptions or corrections, such as tests of normality and adjustment for multiple comparisons
☐	☒	A full description of the statistical parameters including central tendency (e.g. means) or other basic estimates (e.g. regression coefficient) AND variation (e.g. standard deviation) or associated estimates of uncertainty (e.g. confidence intervals)
☐	☒	For null hypothesis testing, the test statistic (e.g. *F*, *t*, *r*) with confidence intervals, effect sizes, degrees of freedom and *P* value noted<br>*Give P values as exact values whenever suitable.*
☒	☐	For Bayesian analysis, information on the choice of priors and Markov chain Monte Carlo settings
☒	☐	For hierarchical and complex designs, identification of the appropriate level for tests and full reporting of outcomes
☒	☐	Estimates of effect sizes (e.g. Cohen's *d*, Pearson's *r*), indicating how they were calculated

Our web collection on statistics for biologists contains articles on many of the points above.

Software and code

Policy information about availability of computer code

Data collection	Commercial:<br>MC_Rack (MultiChannel Systems) 4.6.2, TCX-Control (MultiChannel Systems) 1.3.4, DLP LightCrafter Control Software (Texas Instruments) 5.0.0, ThorCam 3.5.1 (Thorlabs), Pylon5 5.0.0 (Basler AG), Fisher-Yates random permutation algorithm (custom code).
Data analysis	Commercial: MATALB (Mathworks) 2019b, Igor Pro (WaveMetrics) 6.3.7.2<br>Expectation maximization algorithm (custom code), Fisher-Yates random permutation algorithm (custom code).<br>The code to recreate and visualize the chromatic integration, chromatic grating and local chromatic integration stimuli are available at:<br>https://gin.g-node.org/gollischlab/Khani_and_Gollisch_2021_RGC_spike_trains_chromatic_integration<br>The code to analyze chromatic integration properties of the recorded ganglion cells is available at:<br>https://github.com/gollischlab/ChromaticIntegrationAnalysis

For manuscripts utilizing custom algorithms or software that are central to the research but not yet described in published literature, software must be made available to editors and reviewers. We strongly encourage code deposition in a community repository (e.g. GitHub). See the Nature Research guidelines for submitting code & software for further information.

Data

Policy information about availability of data

All manuscripts must include a data availability statement. This statement should provide the following information, where applicable:

- Accession codes, unique identifiers, or web links for publicly available datasets
- A list of figures that have associated raw data
- A description of any restrictions on data availability

The spike-time data that support the findings of this study are deposited in a public repository at:

https://gin.g-node.org/gollischlab/Khani_and_Gollisch_2021_RGC_spike_trains_chromatic_integration
Images used to construct the models of chromatic integration for natural scenes were taken from: UV/Green Image Databases, available at: https://www.ti.uni-bielefeld.de/html/people/ddiffert/databases_uvg.html

Field-specific reporting

Please select the one below that is the best fit for your research. If you are not sure, read the appropriate sections before making your selection.

- [x] Life sciences
- [ ] Behavioural & social sciences
- [ ] Ecological, evolutionary & environmental sciences

For a reference copy of the document with all sections, see nature.com/documents/nr-reporting-summary-flat.pdf

Life sciences study design

All studies must disclose on these points even when the disclosure is negative.

Sample size	Sample sizes were not statistically predetermined. Multi-electrode-array recordings yield large, yet unpredictable numbers of simultaneously recorded cells (typically 100-200 retinal ganglion cells per experiment), which provides more than sufficient sampling of cells from each retina. For this study we recorded around 3400 retinal ganglion cells from 31 retinas of 19 mice to ensure repeatability of results across animals.
Data exclusions	Recorded units with no clear refractory period and not well-separated cluster of voltage traces were excluded during spike sorting. These are typical criteria to avoid contamination by units that do not match individual cells. In the analysis of chromatic grating stimulus, cells with firing rate <5Hz in PSTH for all contrast combinations were excluded to avoid cells with low signal-to-noise ratio. In the analysis of the local chromatic integration stimulus, cells whose receptive field (RF) midpoint differed by more than with a RF radius from the local stimulus location were excluded to avoid cells for which also the RF surround was strongly activated. The data exclusion criteria are stated in the corresponding Methods sections in the manuscript. None of the mentioned exclusion criteria were pre-established.
Replication	All experiments were repeated multiple time in different animals (31 retinas from 19 mice of either sex) with consistent results. Experimental conditions were designed to ensure consistency between the experiments to guarantee reproducibility of results.
Randomization	Animals were not assigned to groups; thus no randomization was performed. For recordings, stimulus conditions were randomized wherever possible. In the design of the chromatic integration stimulus (local and global version) and the chromatic grating stimulus, the order of the contrasts presented were randomized to reduce effects of adaptation.
Blinding	There was no assignment of animals or data into groups, and blinding was therefore not relevant for our experiments.

Reporting for specific materials, systems and methods

We require information from authors about some types of materials, experimental systems and methods used in many studies. Here, indicate whether each material, system or method listed is relevant to your study. If you are not sure if a list item applies to your research, read the appropriate section before selecting a response.

Materials & experimental systems

n/a	Involved in the study	
x		Antibodies
x		Eukaryotic cell lines
x		Palaeontology and archaeology
	x	Animals and other organisms
x		Human research participants
x		Clinical data
x		Dual use research of concern

Methods

n/a	Involved in the study	
x		ChIP-seq
x		Flow cytometry
x		MRI-based neuroimaging

Animals and other organisms

Policy information about studies involving animals; ARRIVE guidelines recommended for reporting animal research

Laboratory animals	Mice strain C57BL/6, either sex, aged 8-13 weeks. The mice were obtained from Charles River Laboratories Germany and housed at 20-24°C with 50-70% humidity on a 12-h light/dark cycle.
Wild animals	No wild animals were used.
Field-collected samples	No field collected samples were used.
Ethics oversight	The applied harvesting of retinal tissue does not require formal ethical approval, but has undergone a voluntary institutional assessment. All the experiments and procedures conformed to national and institutional guidelines and were approved by the institutional animal care committee of the University Medical Center Göttingen (protocol number T11/35).

Note that full information on the approval of the study protocol must also be provided in the manuscript.
